# Prevalence and distribution of subtypes of *Blastocystis* in Asiatic brush-tailed porcupines (*Atherurus macrourus*), bamboo rats (*Rhizomys pruinosus*), and masked palm civets (*Paguma larvata*) farmed in Hainan, China[Fn FN1]

**DOI:** 10.1051/parasite/2023048

**Published:** 2023-11-02

**Authors:** Wei Zhao, Yun Zhang, Jiaqi Li, Guangxu Ren, Yu Qiang, Yuan Wang, Xiuyi Lai, Sheng Lei, Rui Liu, Yuankun Chen, Huicong Huang, Wenting Li, Gang Lu, Feng Tan

**Affiliations:** 1 Department of Parasitology, Wenzhou Medical University Wenzhou 325035 China; 2 Key Laboratory of Tropical Translational Medicine of Ministry of Education, NHC Key Laboratory of Tropical Disease Control, Hainan Medical University-The University of Hong Kong Joint Laboratory of Tropical Infectious Diseases, Department of Pathogenic Biology, Hainan Medical University Haikou 571199 China; 3 Department of Infectious and Tropical Diseases, the Second Affiliated Hospital of Hainan Medical University Haikou 570100 China

**Keywords:** *Blastocystis* sp., Zoonotic, Subtypes, Farm animals, Hainan (China)

## Abstract

*Blastocystis* sp. is an important gastrointestinal parasite with global distribution, prevalent in humans, farmed animals, and wildlife. Therefore, this study aimed to investigate the prevalence and genetic diversity of *Blastocystis* sp. in Asiatic brush-tailed porcupines (*Atherurus macrourus*), bamboo rats (*Rhizomys pruinosus*), and masked palm civets (*Paguma larvata*) in Hainan Province, China. A total of 900 fecal samples were collected from three farmed animal species including 257 porcupines, 360 rats, and 283 civets. Genomic DNA was extracted from each fecal sample and *Blastocystis* sp. was detected by PCR at the small subunit ribosomal RNA (*SSU* rRNA) gene. A phylogenetic tree was constructed using the maximum likelihood method. *Blastocystis* sp. was detected in 47 (5.2%) fecal samples: 12 (4.7%) Asiatic brush-tailed porcupines, 8 (2.2%) bamboo rats, and 27 (9.5%) masked palm civets. Three known *Blastocystis* sp. subtypes, including ST1, ST4, ST5, and one unnamed subtype (unST), were found in one, 19, 26, and one animal, respectively. Subtypes ST4 and unST were detected in porcupines, ST4 in rats, and ST1 and ST5 in civets. Our results suggest that the three farmed animal species reported in this study could serve as reservoirs for potentially zoonotic *Blastocystis* sp. subtypes and transmit this parasite to humans, other farmed animals, and wildlife.

## Abstract (fr)

*Blastocystis* sp. est un parasite gastro-intestinal important avec une distribution mondiale, répandu chez les humains, les animaux d’élevage et la faune. Par conséquent, cette étude visait à étudier la prévalence et la diversité génétique de *Blastocystis* sp. chez les athérures à longue queue (*Atherurus macrourus*), les rats des bambous (*Rhizomys pruinosus*) et les civettes masquées (*Paguma larvata*) dans la province de Hainan, en Chine. Au total, 900 échantillons fécaux ont été collectés sur ces trois espèces animales d’élevage dont 257 athérures, 360 rats et 283 civettes. L’ADN génomique a été extrait de chaque échantillon fécal et *Blastocystis* sp. a été détecté par PCR au niveau du gène de la petite sous-unité de l’ARN ribosomal. Un arbre phylogénétique a été construit en utilisant la méthode du maximum de vraisemblance. *Blastocystis* sp. a été détecté dans 47 (5,2 %) échantillons fécaux : 12 (4,7 %) athérures, 8 (2,2 %) rats et 27 (9,5 %) civettes. Trois sous-types de *Blastocystis* sp., dont ST1, ST4, ST5 et un sous-type sans nom (unST), ont été trouvés respectivement chez 1, 19, 26 et 1 animal. Les sous-types ST4 et unST ont été détectés chez les athérures, ST4 chez les rats et ST1 et ST5 chez les civettes. Nos résultats suggèrent que les trois espèces animales d’élevage concernées par cette étude pourraient servir de réservoirs à des sous-types potentiellement zoonotiques de *Blastocystis* sp. et transmettre ce parasite aux humains, à d’autres animaux d’élevage et à la faune.

## Introduction

*Blastocystis* sp. is a ubiquitous gut parasite that causes intestinal infections in humans and almost all animal species [12, 26]. The pathogenicity of *Blastocystis* sp. is controversial, and *Blastocystis* sp. infection is generally asymptomatic. Nonetheless, symptoms such as diarrhea, abdominal pain, weight loss, and bloating may occur in some patients [2]. Zoonotic, food and waterborne, and human-to-human transmission are the main routes through which *Blastocystis* sp. is transmitted [32]. In most instances, infection occurs *via* the fecal-oral route by drinking or eating the *Blastocystis* sp. cysts in contaminated food or water [21]. Identifying the source of *Blastocystis* sp. infections is key in preventing and controlling the spread of the parasite. However, there are limited data on the role of farmed animals in transmitting *Blastocystis* sp. to humans.

Molecular methods are widely used to detect *Blastocystis* sp. and are considered useful for identifying *Blastocystis* sp. reservoirs or hosts [29]. To date, approximately 40 *Blastocystis* sp. subtypes infecting humans and animals have been reported [12, 26, 30]. Rodents and carnivores may play important roles in the ecology and transmission of several *Blastocystis* sp. subtypes, including ST1–ST5, ST7, ST8, ST10, ST13, and ST17 (in rodents) and ST1–ST5, ST7, ST10, and ST14 (in carnivores) [12]. Except for ST13 and ST17, all the above *Blastocystis* sp. subtypes infecting rodents and carnivores have been detected in humans, suggesting that these animals are potential reservoirs of *Blastocystis* sp. [12].

Asiatic brush-tailed porcupines (*Atherurus macrourus*), bamboo rats (*Rhizomys pruinosus*), and masked palm civets (*Paguma larvata*) have recently been assessed for the IUCN Red List of Threatened Species, and in China, they are commonly farmed for food in Hainan Province. However, the role of these animals for *Blastocystis* sp. is not well understood. Therefore, this study used PCR to investigate the prevalence and genetic diversity of *Blastocystis* sp. in Asiatic brush-tailed porcupines, bamboo rats, and masked palm civets in Hainan Province to assess their zoonotic potential.

## Material and methods

### Ethics statement

This study was approved by the Research Ethics Committee and the Animal Ethical Committee of Wenzhou Medical University. Only excreted feces from the study animals were analyzed. No animal was injured during the research.

### Sample collection

**Figure 1:**
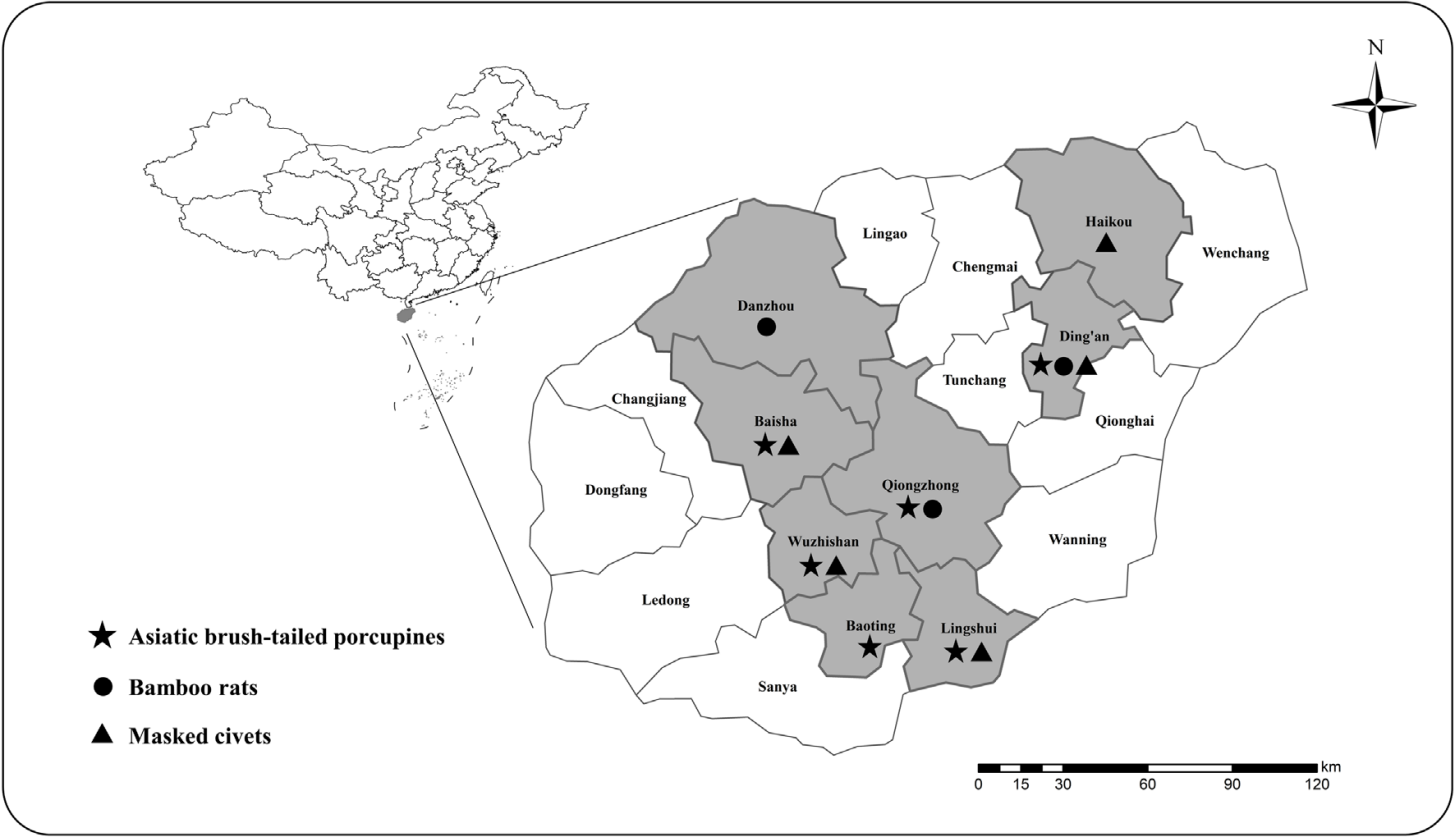
A map depicting the sampling sites for collecting fecal samples from Asiatic brush-tailed porcupines, bamboo rats and masked palm civets in Hainan Province, China.

A total of 900 fecal samples were collected from 257 Asiatic brush-tailed porcupines, 360 bamboo rats, and 283 masked palm civets farmed in Hainan Province between December 1, 2017, and March 31, 2021 (Table 1). Asiatic brush-tailed porcupine samples were obtained from 6 farms in Baisha, Baoting, Lingshui, Wuzhishan, Dingan, and Qiongzhong; those of bamboo rats were sampled from 3 farms in Danzhou, Dingan, and Qiongzhong; and those of masked palm civets were sampled from 5 farms in Baisha, Dingan, Haikou, Lingshui, and Wuzhishan (Fig. 1). To minimize duplicate sampling, only one fecal sample (~15 g) was collected in each animal cage (each with 1 to 3 animals). Approximately 30–50% of animals on each farm were sampled. The farms were selected based on the willingness of the owners to participate in the study. All farms had a relatively good standard of environmental sanitation where animals are provided with clean tap water for drinking, 1 to 3 animals were kept in individual cages with minimal contact with other species. The management of animals on all fourteen farms was similar. Fresh fecal samples were collected in labeled sterile plastic bags and transported to the Key Laboratory of Tropical Translational Medicine of the Ministry of Education (Hainan Medical University) in containers packed with ice packs within 24 h and stored at 4 °C for processing within 12 h. None of the animals showed diarrhea at the time of sample collection.

**Table 1:** Prevalence and distribution of STs of *Blastocystis* in the three farmed animals in Hainan Province, China.

Hosts	Infection rate (%) (No. positive/No. examined)	STs (*n*)
Asiatic brush-tailed porcupines (*Atherurus macrourus*)		
Baisha	1.1 (1/89)	ST4 (1)
Baoting	8.2 (4/49)	ST4 (4)
Dingan	0 (0/19)	–
Lingshui	1.7 (1/59)	ST4 (1)
Qiongzhong	0 (0/11)	–
Wuzhishan	20 (6/30)	ST4(5), unST (1)
Subtotal	4.7 (12/257)	ST4(11), unST (1)
Bamboo rats (*Rhizomys pruinosus*)		
Danzhou	7.0 (8/114)	ST4 (8)
Dingan	0 (0/10)	–
Qiongzhong	0 (0/236)	–
Subtotal	2.2 (8/360)	ST4 (8)
Masked Palm civets (*Paguma larvata*)		
Baisha	0 (0/72)	–
Dingan	0 (0/108)	–
Haikou	0 (0/32)	–
Lingshui	48.2 (27/56)	ST5 (26), ST1 (1)
Wuzhishan	0 (0/15)	–
Subtotal	9.5 (27/283)	ST5 (26), ST1 (1)
Total	5.2 (47/900)	ST5 (26), ST4 (19), ST1 (1), unST (1)

### DNA extraction

Each fecal sample was sieved, and the filtrates were washed three times with distilled water and then centrifuged for 10 min at 1500 ×*g* to concentrate the parasites. Genomic DNA was extracted from 180–200 mg of clean fecal samples using a QIAamp DNA Stool Mini Kit (QIAGEN, Hilden, Germany), according to the manufacturer’s instructions. The extracted DNA was eluted in 200 μL of AE buffer and stored at −20 °C prior to PCR analysis.

### PCR amplification

The bamboo rats were identified to the species level by amplification of a 421-bp region of the cytb gene from fecal DNA using PCR [33]. To detect *Blastocystis* sp*.*, an approximately 500 base pair fragment of the *SSU* rDNA gene was amplified by PCR as described by Santin *et al.* [29]. TaKaRa Taq DNA Polymerase (TaKaRa Bio Inc., Tokyo, Japan) was used for all PCR amplifications. Negative controls were included in all PCR tests for quality control. The PCR products were subjected to 1.5% agarose gel electrophoresis and were visualized on a Gel Doc EZ UV-gel imaging system (Bio-Rad Inc., USA). The gel was stained with GelRed (Biotium Inc., Hayward, CA) to aid in visualization.

### Nucleotide sequencing and analysis

The PCR products of interest were sequenced by Sangon Biotech Co., Ltd (Shanghai, China). Sequence accuracy was confirmed by two-directional sequencing and further sequencing of PCR products for some DNA samples where necessary. Sequences of each strand were edited and aligned using DNASTAR Lasergene EditSeq v7.1.0 (http://www.dnastar.com/) and Clustal X v2.1 (http://www.clustal.org/) tools. Reference sequences were downloaded from the GenBank.

### Phylogenetic analysis

Mega 7 was used to produce a phylogenetic tree for nucleotide sequences using the maximum likelihood method. One thousand bootstrap replicates were used to test the phylogenetic tree’s reliability and the statistical support for the topology. Evolutionary distances were calculated using the Tamura-3 parameter model [16].

### Nucleotide sequence accession numbers

The novel nucleotide sequence of *Blastocystis* ST5 obtained in this study was deposited in the GenBank database under accession number: OP132525. Meanwhile, all the other sequences which similarity of 100% with sequences available in GenBank obtained here were submitted to GenBank under accession number: OR593325 for ST1, OR593326 to OR593328 for ST4, OR593329 for unST, and OR593330 to OR593339 for ST5.

### Statistical analyses

Data were analyzed using SPSS version 22.0 (SPSS Inc., Chicago, IL, USA). The chi-square test at 95% confidence interval was used to compare the prevalence of *Blastocystis* sp. among the different animal species. *P* < 0.05 was considered statistically significant.

## Results

### Infection rates of *Blastocystis* sp.

*Blastocystis* sp. was detected in 47 (5.2%) of the 900 fecal samples from 27 (9.5%) masked palm civets, 12 (4.7%) Asiatic brush-tailed porcupines, and 8 (2.2%) bamboo rats (Table 1). Notably, the differences in the prevalence rates of *Blastocystis* sp. among the three species and across the farms sampled were statistically significant (χ^2^ = 17.4, df = 2, *p* < 0.001). Positive fecal samples of Asiatic brush-tailed porcupines were from four of the six farms surveyed, with the highest infection rate (20.0%) in Wuzhishan, followed by Baoting, Lingshui, and Baisha (Table 1). Positive fecal samples of bamboo rats and masked palm civets were from one out of all the farms surveyed. The prevalence of *Blastocystis* sp. in bamboo rats in Danzhou farm was 7.0%, and 48.2% in masked palm civets in Lingshui farm (Table 1).

### Sequencing of PCR amplicons

PCR and sequencing analysis of cytb amplicons showed that all the bamboo rats were *Rhizomys pruinosus* and all cytb gene sequences had 100% identity with the reference sequence KY754146*.* Meanwhile all the 47 PCR amplicons were sequenced to determine *Blastocystis* sp. subtypes. Nucleotide sequence analysis revealed the presence of three distinct previously reported subtypes (ST1, ST4, and ST5) and one unnamed subtype (unST); there were no mixed infections detected. Among them, ST5 was the most common subtype detected at 55.3% (26/47), followed by ST4 at 40.4% (19/47). The prevalence of ST1 and the unST were each at 2.1% (1/47). While ST4 was detected in both Asiatic brush-tailed porcupines and bamboo rats, ST1 and ST5 were detected in masked palm civets only, and the unST was detected in one Asiatic brush-tailed porcupine (Table 1).

### Genetic diversity of *Blastocystis* subtypes

**Figure 2:**
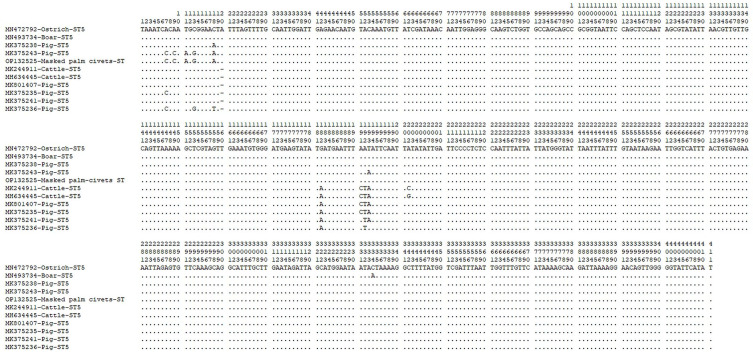
Sequence variation in the small subunit (SSU) rRNA gene among of *Blastocystis* sp. ST5. SSU rRNA sequences of ST5 identified in this study were aligned. MN472792 as the reference sequence.

**Figure 3:**
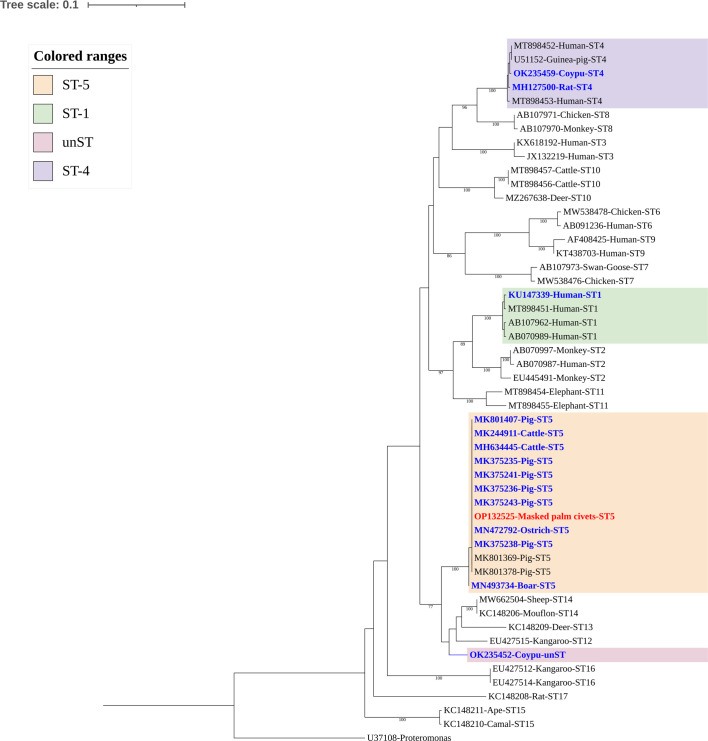
Phylogenetic relationship of *Blastocystis* sp. STs based on SSU rRNA gene sequences. Clustal X and Mega 7 software were used to calculate genetic distance and build the phylogenetic tree. The phylogenetic tree was constructed using the maximum likelihood method, based on the evolutionary distances calculated by the Tamura-3 parameter model; all positions containing gaps and missing data were eliminated. Bootstrap values were obtained using 1,000 replicates. The blue and red fonts represent the known and novel sequences, respectively identified in this study.

Except for one ST5 sequence (OP132525), which had a 99.8% identity with MK375243 isolated from a pig in Yunan Province, the rest of the 46 sequences have been previously described. The 25 ST5 displayed 100% genetic identity with previously reported subtypes: MN472792 (*n* = 7), MK244911 (*n* = 5), MK375238 (*n* = 3), MH634445 (*n* = 3), MK375235 (*n* = 2), MN493734 (*n* = 1), MK375243 (*n* = 1), MK375236 (*n* = 1), MK375241 (*n* = 1) and MK801407 (*n* = 1). A sequence alignment analysis of these ST5 sequences shows that there were 12 polymorphic sites among them (Fig. 2). A total of 19 nucleotide sequences were identified as ST4, with 13 of these from Asiatic brush-tailed porcupine (*n* = 5) and bamboo rats (*n* = 8) displaying 100% identity with *Blastocystis* sp. subtypes isolated from *Rattus norvegicus* in Japan (MH127500). The remaining six ST4 sequences were isolated from Asiatic brush-tailed porcupine and displayed 100% genetic identity with *Blastocystis* sp. subtype isolated from coypu in China (OK235459). ST1 and unST displayed an identity of 100% with a *Blastocystis* sp. subtype isolated in human from Mexico (KU147339) and a coypu from Henan Province (OK235452), respectively. Overall, nucleotide sequences of *Blastocystis* sp. subtypes obtained from this study clustered together with known subtypes in the phylogenetic tree except for the sequence of the unST, which fell into a distinct evolutionary branch (Fig. 3).

## Discussion

In the present study, *Blastocystis* sp. was detected in 5.2% (47/900) of animals, including 4.7% (12/257) Asiatic brush-tailed porcupine, 2.2% (8/360) bamboo rats, and 9.5% (27/283) masked palm civets sampled (Table 1). No study has reported on *Blastocystis* sp. in Asiatic brush-tailed porcupines and masked palm civets. Also, only one study has reported *Blastocystis* sp. infection in bamboo rats [31]. Unlike the low infection rate of 2.2% in bamboo rats sampled in this study, the single previous study on the same animal species from this area reported a higher prevalence rate of 4.58% [31]. Analysis of previous studies on the prevalence of *Blastocystis* sp. in wild and farmed animal species such as rodents and carnivores indicate geographical variations in infection rates, ranging from 3.1–100% [1, 3–10, 11, 13–15, 17–20, 22–25, 27, 28, 31, 34–36]. The differences in infection rates are possibly due to host variation in different regions. The prevalence of *Blastocystis* sp. in the two farmed rodents investigated in the present study was lower compared to other farmed rodents, such as in *Myocastor coypus* (14.3%) and in flying squirrels (30.4%) in China [9, 34], and in guinea pigs (35.4%) from Ecuador [11]. In addition to the animal species, the sample size, the health of the animals, the environment, geographical location, and the type of detection method may explain the variability in the prevalence rates. It is worth noting that only one study was done in 12 out of the 15 countries for rodents and seven out of eight countries for carnivores with extremely small sample sizes [1, 3–10, 11, 13–15, 17–20, 22–25, 27, 28, 31, 34–36]. The presence of *Blastocystis* sp. in rodents and carnivores is a public health concern, requiring large-scale investigations to assess their role in transmitting this parasite to humans.

Further, this study uncovered three known *Blastocystis* sp. subtypes (ST1, ST4, and ST5) and one unnamed subtype (unST). ST4 and ST5 were the predominant *Blastocystis* sp. subtypes (95.7%) detected in this study. Previous studies show that ST4 and ST5 are circulating in bamboo rats in the Hunan Province [31]. Moreover, *Blastocystis* sp. surveys in other studies have reported several other *Blastocystis* sp. subtypes, including potential zoonotic subtypes in rodents, coati, pandas, foxes, wolves, dogs, wild cats, and raccoons [1, 3–10, 11, 13–15, 17–20, 22–25, 27, 28, 31, 34–36]. The prior data show that ST5 is rare in carnivores, which is significantly different from the results of this study and reflects regional characteristics.

ST5 was the most prevalent *Blastocystis* sp. subtype in this study (55.3%). This subtype has previously been detected in multiple animal hosts, mostly pigs, with a 1.64% infection rate reported in human samples worldwide [26]. The ST5 prevalence rate in farmed masked palm civets suggests zoonotic transmission of this parasite from these animals to humans, pigs, and other animals, or, conversely, the ST5 harbored in the masked palm civets may also come from pigs or humans. Almost 95.0% of *Blastocystis* sp. detected in bamboo rats and porcupines were ST4. The global *Blastocystis* sp. ST4 prevalence in humans is 5.9%, although in Europe it is responsible for 19.8% of the cases reported [26]. This subtype has also been detected in rodents, birds, several deer species, and other wild mammals worldwide, indicating that it has a wide host range [27]. Given the prevalence of *Blastocystis* sp. ST4 in rodents (>17 rodent species), the subtype has possibly adapted to infect this type of host. Since most rodent species are peridomestic, they are considered potential sources of human *Blastocystis* sp. infections, needing extensive investigation. Although ST1 was only detected in one sampled animal (2.1%), the subtype is an important public health parasite because it is the second most common *Blastocystis* sp. subtype infecting humans and has been detected in animals worldwide [12, 26]. The unST detected in the present study has 92.4% identity with ST14. Given that this unST has been detected in a coypu in Henan [9], it potentially infects multiple hosts, including coypu and Asiatic brush-tailed porcupine.

## Conclusion

This is the first report on the prevalence and genetic diversity of *Blastocystis* sp. in Asiatic brush-tailed porcupines, bamboo rats, and masked palm civets in Hainan Province. Our results indicate circulation of three known *Blastocystis* sp. subtypes (ST1, ST4, and ST5) and an unnamed *Blastocystis* sp. subtype in the animals mentioned above. These findings could provide a basis for preventing and controlling *Blastocystis* sp. infections in humans and the farmed animals in this region.

## Conflict of interest

The authors do not have a commercial or other association that represents a conflict of interest.

## Funding

This work was supported by the Department of Education Scientific Research Project of Zhejiang (Y202249687), National Natural Science Foundation of China (82060375), Research project of Hainan academician innovation platform (YSPTZX202004), Hainan talent development project (SRC200003), Major Science and Technology Program of Hainan Province (ZDKJ202003), Open Foundation of Key Laboratory of Tropical Translational Medicine of Ministry of Education, Hainan Medical University (2020TTM004), High level talents project of Hainan Natural Science Foundation (822RC695) and, Science and Technology Plan of Hainan Province (Hainan Province Clinical Medical Center). The funding sponsors had no role in study design, data collection and analysis, decision to publish, or preparation of the manuscript.
